# Whole Animal Genome Sequencing: user-friendly, rapid, containerized pipelines for processing, variant discovery, and annotation of short-read whole genome sequencing data

**DOI:** 10.1093/g3journal/jkad117

**Published:** 2023-05-27

**Authors:** Jonah N Cullen, Steven G Friedenberg

**Affiliations:** Department of Veterinary Clinical Sciences, College of Veterinary Medicine, University of Minnesota, 1352 Boyd Ave, Saint Paul, MN 55108, USA; Department of Veterinary Clinical Sciences, College of Veterinary Medicine, University of Minnesota, 1352 Boyd Ave, Saint Paul, MN 55108, USA

**Keywords:** whole genome sequencing, pipeline, variants

## Abstract

Advancements in massively parallel short-read sequencing technologies and the associated decreasing costs have led to large and diverse variant discovery efforts across species. However, processing high-throughput short-read sequencing data can be challenging with potential pitfalls and bioinformatics bottlenecks in generating reproducible results. Although a number of pipelines exist that address these challenges, these are often geared toward human or traditional model organism species and can be difficult to configure across institutions. Whole Animal Genome Sequencing (WAGS) is an open-source set of user-friendly, containerized pipelines designed to simplify the process of identifying germline short (SNP and indel) and structural variants (SVs) geared toward the veterinary community but adaptable to any species with a suitable reference genome. We present a description of the pipelines [adapted from the best practices of the Genome Analysis Toolkit (GATK)], along with benchmarking data from both the preprocessing and joint genotyping steps, consistent with a typical user workflow.

## Introduction

High-throughput sequencing (HTS) has revolutionized the field of genetics and helped usher in today's modern genomic era. Continued advances in sequencing technologies coupled with decreased costs have empowered researchers to generate massive amounts of short-read sequencing data, particularly for whole genome sequencing (WGS) studies. WGS projects may range from single individuals with a phenotype of interest to entire cohorts, broadening our ability to answer biological questions at population-level scales. In addition to large efforts in human medicine (Auton *et al.* 2015), the revolution in HTS has been equally fruitful in veterinary medicine, where research groups across the globe have generated WGS data from tens of thousands of animals across a range of species (Rubin *et al.* 2012; Daetwyler *et al.* 2014; Jagannathan *et al.* 2019; Ostrander *et al.* 2019; Buckley *et al.* 2020; Wang *et al.* 2020; Durward-Akhurst *et al.* 2021).

Working with WGS data, however, is not always straightforward. The processing of WGS data is often inaccessible to many researchers and relatively nonstandard. For years, the Broad Institute has been at the vanguard in developing open-source tools [e.g. Genome Analysis Toolkit (GATK)], analysis pipelines, and best practices geared for human WGS projects (der Auwera and O’Connor 2020). Reconfiguring GATK pipelines for nonhuman WGS samples can be a challenging and potentially error-prone endeavor when scaled to dozens or hundreds of samples. For example, generating genome and alignment indices, variant calling intervals, and preprocessing FASTQ files to unmapped binary alignment map (uBAM) files (i.e. the expected inputs for the GATK germline short variant discovery pipeline) require substantial bioinformatics expertise and familiarity with many command-line-based tools. Numerous bioinformatics pipelines exist for processing and analyzing WGS data (Evani *et al.* 2012; Karczewski *et al.* 2014; Guo *et al.* 2015; Menon *et al.* 2016; Elshazly *et al.* 2017; Causey *et al.* 2018; Ahmed *et al.* 2021) with varying degrees of usability, accessibility, and adaptability, resulting in a spectrum of potential challenges. These challenges may be exacerbated when setting up these pipelines on a remote high-performance computing (HPC) environment using a job scheduler. Moreover, many of these pipelines do not utilize container software or use Docker (Merkel 2014) as a way of standardizing their bioinformatics workflow. Container software, like Docker, provides lightweight, portable computational environments that can package data (e.g. reference genomes) and software along with their dependencies, enabling consistent and reproducible computation across computing environments. However, Docker is often incompatible with HPC environments found at many academic institutions. Given the potential for pipeline output discrepancies due to differences in software versions and dependencies, the use of containers in processing WGS data could be regarded as essential. Without containers, it is difficult to ensure reproducibility necessary for comparing and synthesizing results across studies or institutions. The enforced reproducibility provided by containers ensures that pipeline outputs are consistent regardless of where the output was generated. Thus, the use of container software specifically designed for scientific computing such as Singularity/Apptainer (Kurtzer *et al.* 2017), in conjunction with a workflow management system (e.g. Snakemake (Mölder *et al.* 2021)) for WGS pipelines, is a central component to ensuring reproducibility, adaptability, and transparency.

To address these hurdles, we developed scalable, adaptable, open-source, containerized, Snakemake-based pipelines tailored for the veterinary community under the umbrella term Whole Animal Genome Sequencing (WAGS) (https://github.com/jonahcullen/wags). These pipelines, which are meant to be run on a high-performance Linux-based cluster, employ best practices championed by GATK developers while lowering barriers often faced by animal researchers seeking to analyze WGS data. WAGS consists of 3 pipelines. The first pipeline processes raw FASTQ files into genomic variant call format (GVCF) files and is capable of processing samples split across flow cells or lanes. The second pipeline takes multiple GVCF files as inputs and performs joint genotype calling and variant annotation. And the third pipeline compares variants in a specific sample to variants in a larger variant call file (VCF) database to enable the identification of unique variants in a particular animal.

## Methods

**Fig. 1.**
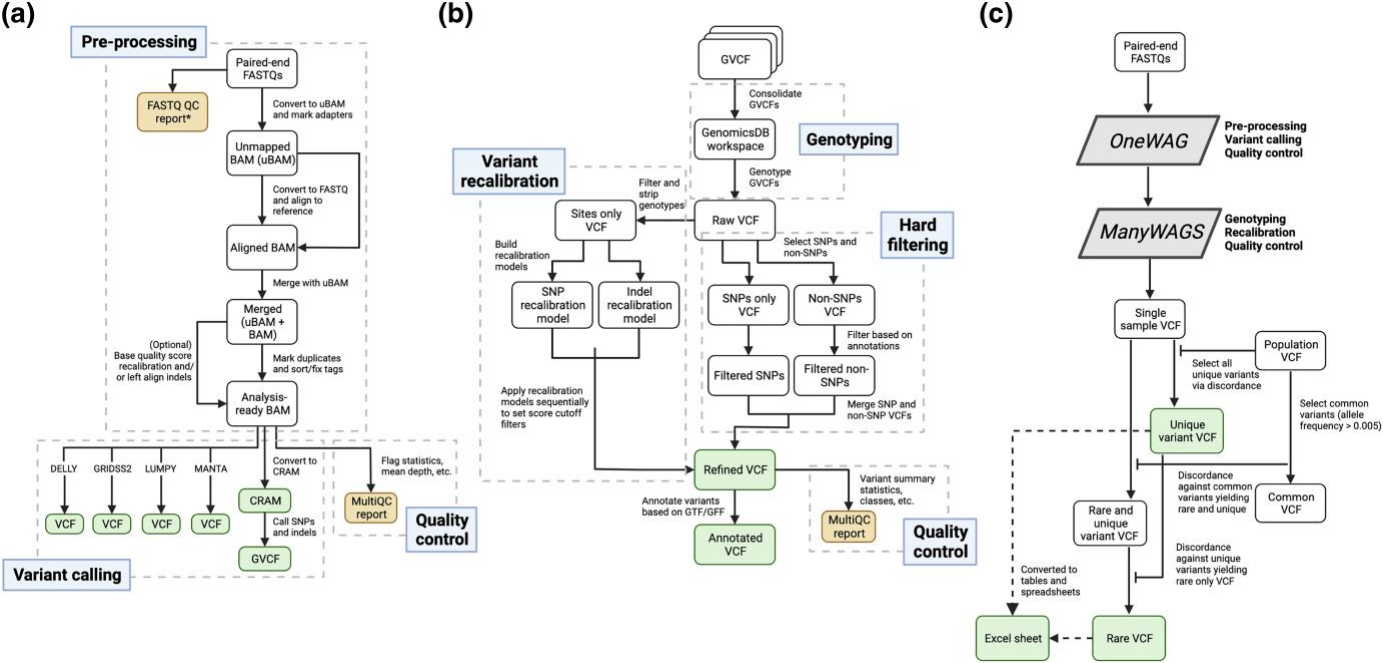
Overview of the WAGS pipelines. a) *OneWAG* for single-sample variant calling starting from paired-end FASTQ files. b) *ManyWAGS* for joint genotyping sample cohorts. c) Identification of unique and rare variants from a single sample compared to a multisample variant call set (*OnlyWAG*). For *OnlyWAG*, the single-sample VCF uses 2 sets of call sets: (1) unique variants and (2) variants that are considered rare (allele frequency < 0.005) and unique. The unique variants from the latter are then filtered out by comparison to the unique variant call set, yielding a set of unique *and* rare variants from the input sample. Created with BioRender.com.

**Fig. 2.**
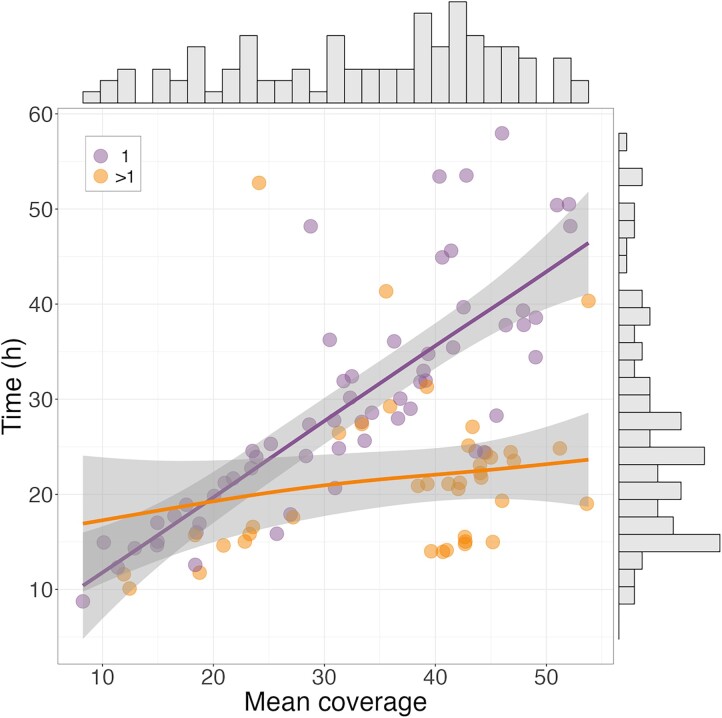
Processing times from FASTQ files through GVCF using the *OneWAG* pipeline. Samples with a single FASTQ pair (*n* = 60, purple) and greater than 1 pair (*n* = 40, orange) are plotted by mean depth with generalized additive model (GAM) smooth lines.

**Fig. 3.**
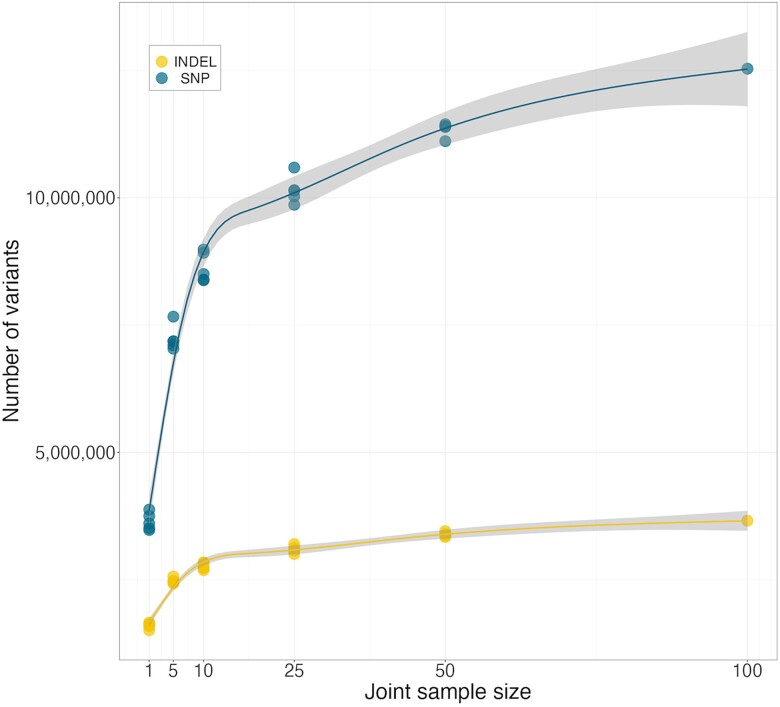
SNP and indel counts at increasing cohort sizes. Cohorts were randomly selected and processed with *ManyWAGS* 5 times for each indicated size (1, 5, 10, 25, and 50) sans 100.

WAGS consists of 3 pipelines for (1) processing raw short-read FASTQ files into GVCF files (*OneWAG*, Fig. 1a), (2) joint genotyping and annotating variants (*ManyWAGS*, Fig. 1b), and (3) identifying private variants in a single sample (*OnlyWAG*, Fig. 1c). The latter pipeline, *OnlyWAG*, combines aspects of *OneWAG* and *ManyWAGS*, generating the same outputs from *OneWAG* in addition to a table of private and rare variants compared to a larger population of animals. All 3 pipelines are Snakemake-based (Mölder *et al.* 2021) and utilize a Singularity container (Kurtzer *et al.* 2017) bundling all required software and reference files for select species (e.g. dog, cat, and horse). Additionally, log files documenting the processing of each step for all 3 pipelines are saved in a designated and structured logs directory, facilitating an understanding of processing errors or analysis of edge cases (e.g. samples with exceedingly high duplication rates). For species not already available in the container, all required inputs (e.g. reference index and dictionary, Burrows-Wheeler Alignment tool (BWA) indices (Li and Durbin 2009)) can be generated from a reference sequence in FASTA format using a separate script *prep_custom_ref.py*. WAGS is designed to be run on an HPC cluster using the Slurm workload manager. Currently, only Slurm is supported (https://github.com/Snakemake-Profiles/slurm) with future plans to include additional schedulers (e.g. Load Sharing Facility, Portable Batch System, and Sun Grid Engine).

### OneWAG

#### Input and setup

To process WGS data from a single sample, a plain text file containing the sample name, the breed (optional), and the prefix of paired-end FASTQ data files is required. This text file is used to generate the pipeline inputs as well as a job script to be submitted to the scheduler. The meta file may contain 1 or more samples to process with 1 sample per row, producing pipeline inputs and submission scripts organized by breed and sample name. There are no limitations on the number of FASTQ files associated with a given sample (e.g. sequenced across multiple lanes or separate flowcells) as long as they share a common portion of the FASTQ file names. Required arguments and various options are described in the documentation (https://github.com/jonahcullen/wags).

#### FASTQ to GVCF

The data processing steps implemented in *OneWAG* are adapted from GATK Best Practice guidelines (der Auwera and O’Connor 2020) and rely almost entirely on these methods unless otherwise noted. *OneWAG* begins by simultaneously assessing FASTQ quality via FastQC (Andrews 2010) and converting paired FASTQ files to a uBAM file (*FastqToSam*). This enables the attachment of user-defined metadata (e.g. sample name) or metadata parsed from the FASTQ identifier (e.g. flowcell ID, lane number) as part of the initial setup. Adapters are then marked (*MarkIlluminaAdapters*) prior to converting the uBAM back to FASTQ, and alignment to a reference genome is performed with BWA (Li and Durbin 2009); these steps are piped together with *view* from SAMtools (Danecek *et al.* 2021) to produce the aligned binary alignment map (BAM) file. The aligned BAM is then merged with the uBAM (*MergeBamAlignment*), retaining the added metadata. The next steps include marking duplicate reads (*MarkDuplicates*), sorting (*SortSam*), adding tags (*SetNmMdAndUqTags*), and generating a BAM index file. Optionally, indels may be left aligned (*LeftAlignIndels*). Depending on the availability of known variant (SNP and indel) positions for the species, base quality score recalibration (BQSR) may be performed. For select species, known variant position files are included within the container and may be utilized. Users may also supply their own known variant position files if desired. BQSR is conducted in parallel using chromosome- or contig-scale intervals by building a recalibration model per interval (*BaseRecalibrator*), combining recalibration reports across intervals (*GatherBQSRReports*), and adjusting the quality score for each base using the combined recalibration report per interval (*ApplyBQSR*). The recalibrated BAM files are gathered across intervals to create a final analysis-ready BAM and index. If BQSR is not desired, the BAM produced following marking duplicates, sorting, and adding tags is considered the final BAM for downstream analysis. Whether or not a recalibration step is included, a compressed BAM (CRAM) and index are generated to minimize data storage requirements (*PrintReads*).

SNPs and indels are called in parallel by first creating an interval list by splitting the reference at stretches of missing base calls (Ns) (tunable with default of maximum 50 Ns tolerated before splitting) (*ScatterIntervalsByNs*; Picard tools, http://broadinstitute.github.io/picard/). The initial interval list is then split into sets (tunable with default of 50 interval sets) of approximately equal size (*SplitIntervals*) over which variants are called in parallel (*HaplotypeCaller*), producing a GVCF for each set. GVCF files for each interval set are then gathered (*MergeVcfs*), resulting in a sample-level GVCF and index, ready for joint genotyping by *ManyWAGS*.

BAM files are also used as inputs to call structural variants (SVs). Due to the difficulty in identifying SVs from short-read sequencing data and the variability between SV calling algorithms, *OneWAG* utilizes 4 well-characterized callers: DELLY (Rausch *et al.* 2012), GRIDSS2 (Cameron *et al.* 2017, 2021), Smoove (Pedersen *et al.* 2020), and Manta (Chen *et al.* 2016). Smoove uses LUMPY (Layer *et al.* 2014) and svtyper (Chiang *et al.* 2015) to identify and genotype SVs. Users may decide which outputs to consider for downstream analysis.

#### Quality control

In addition to the FastQC (Andrews 2010) walker described above, *OneWAG* also provides output from SAMtools’ *flagstat* (Danecek *et al.* 2021) to count alignment types, GATK's *AnalyzeCovariates* to assess BAM recalibration, and Qualimap’s *bamqc* module to provide depth profiles and other parameters (Okonechnikov *et al.* 2016). All QC data and plots are combined into a single, user-friendly report using MultiQC (Ewels *et al.* 2016).

### ManyWAGS

#### Input and setup

To joint genotype GVCF files generated from the *OneWAG* pipeline, a single text file containing sample names and paths of GVCF files is required; an example is provided in the GitHub repository (https://github.com/jonahcullen/wags). Species- or reference-specific configuration files needed for joint genotyping are first generated (*config_joint.py*). Similar to *OneWAG*, all pipeline inputs and a job script are generated to initiate joint genotyping adapted from GATK Best Practice guidelines (der Auwera and O’Connor 2020).

#### GVCF to VCF

To begin processing, the first step is to determine the intervals over which input GVCF files will be processed. There are 2 methods for defining interval boundaries: one of which is based on runs of at least 50 Ns (as in the *OneWAG* pipeline), while the other utilizes midpoints of intergenic regions, thus requiring a reference annotation file such as a gene transfer format (GTF) file. This second option may be particularly useful for nearly complete (i.e. few missing bases) reference genomes. Regardless of the interval boundary method, the resulting intervals are collapsed into larger intervals of an ideal maximum length (default 10 million). This value can be modified; however, this default setting was chosen to balance efficient parallel processing with the number of submissions to the cluster simultaneously (e.g. smaller length intervals will result not only in an increased number of intervals and ultimately faster processing times but also in a higher number of concurrent job submissions, which may be limited by some institutions). GVCF files are then combined into GenomicsDB workspaces via *GenomicsDBImport*, genotyped (*GenotypeGVCFs*), filtered (*VariantFiltration*) by excess heterozygosity, and converted to sites-only VCFs (*MakeSitesOnlyVcf*) for each of the collapsed intervals in parallel. If variant quality score recalibration (VQSR) is enabled, which is strongly encouraged by GATK developers, the sites-only VCFs are combined (*GatherVcfsCloud*), sorted (*SortVcf*), and used as inputs to generate recalibration models for SNPs and indels separately (*VariantRecalibrator*). User-supplied files of known SNP and indel positions are required for this step unless they are already stored within the container. *ApplyVQSR* then filters the collapsed interval VCFs in parallel, where each site is marked as PASS or binned based on user-defined tranche sensitivity. The recalibrated VCFs are combined (*GatherVcfsCloud*) into a complete VCF and indexed (*IndexFeatureFile*). If VQSR is not possible or desired (e.g. lack of known SNP and/or indel sites), hard filtering of SNPs and/or indels may be conducted with default thresholds that may be adjusted. Finally, the complete VCF is annotated with the Ensembl Variant Effect Predictor (VEP) (McLaren *et al.* 2016). For basic VEP annotation, an annotation file (e.g. GTF) is required; these are included within the container for select species but may be added for other species. This last step is also conducted in parallel using the collapsed intervals followed by merging the annotated VCFs, compression, and indexing (Li 2011).

#### Quality control

Summary statistics are collected on the final VCF using both BCFtools’ *stats* and GATK's *CollectVariantCallingMetrics*. Per-interval VEP summaries are also collected, enabling the examination of variant classes and consequences, along with other standard metrics. MultiQC aggregates all QC data and plots into a single, user-friendly report.

### OnlyWAG

The *OnlyWAG* pipeline was designed to identify private or rare variants in a single individual compared to variants in a population of interest generated by *ManyWAGS* or any other VCF. The *OneWAG* pipeline yields an individual GVCF (and all other outputs), which is genotyped using the *ManyWAGS* pipeline to produce a single-sample VCF. The single-sample VCF is compared to the population VCF using GATK's *SelectVariants*, and 2 specific outputs are produced: (1) a VCF containing variants that are unique to the single-sample VCF and (2) a VCF containing variants in the single sample that are rare (tunable with default allele frequency < 0.005) in the population VCF. BCFtools’ *+split-vep* is used to create delimited tables of the unique and rare VCFs, which are then parsed and output as Excel sheets for downstream analysis and easy visualization by end users.

## Results and discussion

### Performance evaluation

WAGS has been utilized and validated for processing and genotyping large cohorts of horses, dogs, and tigers. For example, we have cataloged genetic variants from WGS data of nearly 700 dogs across more than 50 breeds, using 2 canine reference genomes (Lindblad-Toh *et al.* 2005; Hoeppner *et al.* 2014; Wang *et al.* 2021). Ongoing analyses have demonstrated the usefulness of this catalog in identifying genetic variants associated with various Mendelian diseases and traits in our canine companions (Olby *et al.* 2020; Shelton *et al.* 2021; Shelton, Minor, Guo, *et al.* 2022; Shelton, Minor, Vieira, *et al.* 2022). However, unlike humans with the Genome in a Bottle Consortium (Zook *et al.* 2016), we do not have an extensively curated truth set of genetic variants in animal species for validating the accuracy of WAGS. Instead, we report the processing times for *OneWAG* [FASTQ to GVCF, mapped against GCD_1.0 (Wang *et al.* 2021) plus ChrY (GCA_014441545.1, https://www.ebi.ac.uk/ena/browser/view/GCA_014441545.1)] using 100 canine WGS samples as well as *ManyWAGS* for joint genotyping of various cohort sizes.

From a private collection of Illumina short-read, paired-end WGS data from 671 dogs, we randomly selected 100 samples representing 36 breeds that had between 1 and 12 sets of paired FASTQ files, with 60 samples having 1 pair and 40 samples with greater than 1 pair. All FASTQ files used for this analysis have been made publicly available (Supplementary Table 1). As expected, runtime increases with mean coverage, which depends on the number of paired FASTQ inputs (Fig. 2). This is consistent with the understanding that processing multiple smaller inputs in parallel will be more efficient than larger single FASTQ pairs. However, larger runtime differences do not appear to be meaningful unless sample coverage is greater than ∼40×.

To examine joint genotyping runtimes and SNP/indel counts, we randomly selected (without replacement) cohorts of 1, 5, 10, 25, and 50 dogs and ran *ManyWAGS* 5 times per cohort from the 100 samples described above. In addition, the entire cohort of 100 dogs was joint genotyped once. Mean runtimes for each cohort are presented in Table 1. We observed little difference between joint genotyping cohort sizes of 10 and 25; however, the cohort of 50 dogs took nearly 3 times as long. At size 50, the runtimes were more variable, with a standard deviation (SD) of 3.0 and range from 10.1 to 17.3 h. Due to the large number of joint genotyping steps and processing variability on an institutional HPC with shared nodes, runtimes are often impacted by available resources. We also tabulated SNP and indel counts for each cohort size replicate from the summary metrics generated as part of joint genotyping quality control (Fig. 3). Mean SNP counts increased by 98.5% from sizes 1 to 5, with each increase in cohort size adding a smaller percent of SNPs (e.g. a 10.4% increase from a cohort size of 50 to 100 samples) (Table 1). Indel counts displayed a similar pattern with increasing cohort sizes.

**Table 1.** Mean (SD) runtimes for joint genotyping at 6 cohort sizes.

Cohort size	Mean runtime [hours (SD)]	Mean SNP counts	Mean SNP increase (%)	Mean indel counts	Mean indel increase (%)
1	1.6 (0.6)	3,645,205	–	1,600,195	–
5	2.96 (0.4)	7,235,061	98.5	2,478,264	54.9
10	5.00 (0.8)	8,636,849	19.4	2,766,402	11.6
25	5.42 (0.8)	10,155,441	17.6	3,100,775	12.1
50	14.59 (3.0)	11,352,736	11.8	3,390,625	9.3
100	25.24 (NA)	12,535,006	10.4	3,660,728	8.0

Repeated sampling (*n* = 5) at each cohort size except 100, which was only run once. NA = not applicable.

Abundant resources and pipelines tailored for WGS processing and variant discovery are available for humans; however, this is not typically the case for many veterinary species. WAGS was developed in an effort to close this gap and empower veterinary researchers to analyze WGS data in a user-friendly and reproducible manner. As a Snakemake-based, containerized pipeline, FASTQ files may be processed with little user input and configuration, yielding GVCF files with relatively short runtimes. Similarly, genotyping and annotation of moderate-to-large cohorts, a potentially error-prone endeavor, may be accomplished without substantial bioinformatics knowledge. WAGS is open source and open access. We expect that the majority of users will be researchers in veterinary medicine interested in identifying variants associated with a disease or trait of interest in cohorts of public and private WGS data.

## Supplementary Material

jkad117_Supplementary_Data

## Data Availability

The WGS data used to benchmark WAGS are publicly available with Sequence Read Archive accessions listed in Supplementary Table 1. The source code of WAGS is available at https://github.com/jonahcullen/wags. Supplemental material available at G3 online.
