# Supplementary material for: Whole Animal Genome Sequencing: user-friendly, rapid, containerized pipelines for processing, variant discovery, and annotation of short-read whole genome sequencing data
Source: G3 (Bethesda). 2023 May 27;13(8):jkad117. doi: 10.1093/g3journal/jkad117 (PMC10411559; doi:10.1093/g3journal/jkad117)
Supplement: jkad117_Supplementary_Data [file jkad117_supplementary_data.docx]

| **SampleName** | **BioProject** | **Breed** | **Sex** | **BioSample** | **Accession** |
| --- | --- | --- | --- | --- | --- |
| 86397 | PRJNA476342 | West Highland White Terrier | female | SAMN09430727 | SRR7348443 |
| aria | PRJNA937381 | Collie | female | SAMN33428225 | SRR23628687 |
| D00105 | PRJNA503969 | Standard Poodle | male | SAMN10386546 | SRR8163599 |
| D00138 | PRJNA937381 | Pomeranian | male | SAMN33428226 | SRR23628686 |
| D00151 | PRJNA937381 | Boxer | female | SAMN33428227 | SRR23628675 |
| D00619 | PRJNA937381 | Miniature Schnauzer | male | SAMN33428228 | SRR23628664 |
| D00689 | PRJNA937381 | Scottish Deerhound | female | SAMN33428229 | SRR23628639 |
| D00728 | PRJNA937381 | Boxer | female | SAMN33428230 | SRR23628659 |
| D00730 | PRJNA937381 | Boxer | male | SAMN33428231 | SRR23628649 |
| D00914 | PRJNA937381 | Irish Setter | female | SAMN33428232 | SRR23628623 |
| D00991 | PRJNA937381 | Yorkshire Terrier | male | SAMN33428233 | SRR23628609 |
| D00998 | PRJNA937381 | Yorkshire Terrier | male | SAMN33428234 | SRR23628603 |
| D01088 | PRJNA937381 | Scottish Terrier | female | SAMN33428235 | SRR23628685 |
| D01090 | PRJNA937381 | Scottish Terrier | male | SAMN33428236 | SRR23628683 |
| D01104 | PRJNA937381 | Cavalier King Charles Spaniel | female | SAMN33428237 | SRR23628684 |
| D01372 | PRJNA937381 | Labrador Retriever | female | SAMN33428238 | SRR23628682 |
| D01886 | PRJNA937381 | Boxer | female | SAMN33428239 | SRR23628681 |
| D01924 | PRJNA937381 | Miniature Schnauzer | female | SAMN33428240 | SRR23628680 |
| D02119 | PRJNA937381 | Pomeranian | male | SAMN33428241 | SRR23628679 |
| D02133 | PRJNA937381 | Standard Poodle | male | SAMN33428242 | SRR23628678 |
| D02202 | PRJNA937381 | Boxer | missing | SAMN33428243 | SRR23628677 |
| D02335 | PRJNA937381 | Dachshund | male | SAMN33428244 | SRR23628676 |
| D02462 | PRJNA937381 | Standard Poodle | female | SAMN33428245 | SRR23628674 |
| D02627 | PRJNA937381 | Labrador Retriever | female | SAMN33428246 | SRR23628673 |
| D02706 | PRJNA937381 | Golden Retriever | male | SAMN33428247 | SRR23628672 |
| D02726 | PRJNA937381 | Miniature Poodle | female | SAMN33428248 | SRR23628671 |
| D03171 | PRJNA937381 | Miniature Schnauzer | male | SAMN33428249 | SRR23628670 |
| D03297 | PRJNA937381 | Border Collie | male | SAMN33428250 | SRR23628669 |
| D03504 | PRJNA937381 | Pomeranian | female | SAMN33428251 | SRR23628668 |
| D03733 | PRJNA937381 | Miniature Schnauzer | female | SAMN33428252 | SRR23628667 |
| D04488 | PRJNA937381 | Yorkshire Terrier | male | SAMN33428253 | SRR23628666 |
| D04494 | PRJNA937381 | French Bulldog | male | SAMN33428254 | SRR23628665 |
| D04504 | PRJNA937381 | Havanese | male | SAMN33428255 | SRR23628663 |
| D04515 | PRJNA937381 | Australian Shepherd | male | SAMN33428256 | SRR23628662 |
| D04644 | PRJNA937381 | Miniature Schnauzer | female | SAMN33428257 | SRR23628661 |
| D04935 | PRJNA937381 | Miniature Schnauzer | male | SAMN33428258 | SRR23628646 |
| D05160 | PRJNA937381 | Yorkshire Terrier | female | SAMN33428259 | SRR23628645 |
| D05492 | PRJNA937381 | Miniature Schnauzer | female | SAMN33428260 | SRR23628644 |
| D05531 | PRJNA560083 | Labrador Retriever | male | SAMN12567870 | SRR9962250 |
| D05542 | PRJNA937381 | Border Collie | male | SAMN33428261 | SRR23628643 |
| D05935 | PRJNA937381 | Golden Retriever | missing | SAMN33428262 | SRR23628642 |
| D05938 | PRJNA937381 | Golden Retriever | missing | SAMN33428263 | SRR23628641 |
| D05941 | PRJNA937381 | Toy Poodle | female | SAMN33428264 | SRR23628640 |
| D05942 | PRJNA937381 | Golden Retriever | male | SAMN33428265 | SRR23628638 |
| D05948 | PRJNA937381 | Coonhound | male | SAMN33428266 | SRR23628637 |
| D06118 | PRJNA937381 | French Bulldog | male | SAMN33428267 | SRR23628636 |
| D06121 | PRJNA937381 | French Bulldog | female | SAMN33428268 | SRR23628635 |
| D06123 | PRJNA937381 | English Bulldog | male | SAMN33428269 | SRR23628633 |
| D06126 | PRJNA937381 | English Bulldog | female | SAMN33428270 | SRR23628634 |
| D06130 | PRJNA937381 | English Bulldog | female | SAMN33428271 | SRR23628631 |
| D06131 | PRJNA937381 | English Bulldog | male | SAMN33428272 | SRR23628632 |
| D06139 | PRJNA937381 | English Bulldog | female | SAMN33428273 | SRR23628630 |
| D06143 | PRJNA937381 | English Bulldog | female | SAMN33428274 | SRR23628660 |
| D06145 | PRJNA937381 | English Bulldog | female | SAMN33428275 | SRR23628658 |
| D06152 | PRJNA937381 | Irish Wolfhound | female | SAMN33428276 | SRR23628656 |
| D06159 | PRJNA937381 | Irish Wolfhound | female | SAMN33428277 | SRR23628657 |
| D06167 | PRJNA937381 | Irish Wolfhound | female | SAMN33428278 | SRR23628655 |
| D06168 | PRJNA937381 | Irish Wolfhound | female | SAMN33428279 | SRR23628654 |
| D06171 | PRJNA937381 | Bullmastiff | male | SAMN33428280 | SRR23628653 |
| D06173 | PRJNA937381 | Bullmastiff | male | SAMN33428281 | SRR23628652 |
| D06185 | PRJNA937381 | Bullmastiff | female | SAMN33428282 | SRR23628651 |
| D06186 | PRJNA937381 | Bullmastiff | female | SAMN33428283 | SRR23628650 |
| D06225 | PRJNA937381 | Rottweiler | female | SAMN33428284 | SRR23628648 |
| D06235 | PRJNA937381 | Bouvier | female | SAMN33428285 | SRR23628647 |
| D06240 | PRJNA937381 | Whippet | female | SAMN33428286 | SRR23628629 |
| D06254 | PRJNA937381 | Whippet | male | SAMN33428287 | SRR23628627 |
| D06279 | PRJNA937381 | Golden Retriever | female | SAMN33428288 | SRR23628628 |
| D06281 | PRJNA937381 | Golden Retriever | male | SAMN33428289 | SRR23628624 |
| D06282 | PRJNA937381 | Golden Retriever | male | SAMN33428290 | SRR23628622 |
| D06284 | PRJNA937381 | Golden Retriever | male | SAMN33428291 | SRR23628621 |
| D06663 | PRJNA937381 | Cavalier King Charles Spaniel | male | SAMN33428292 | SRR23628620 |
| D06712 | PRJNA937381 | Boxer | male | SAMN33428293 | SRR23628626 |
| D06743 | PRJNA937381 | Dachshund | female | SAMN33444803 | SRR23628625 |
| D06792 | PRJNA937381 | Miniature Schnauzer | male | SAMN33428295 | SRR23628619 |
| D06812 | PRJNA937381 | Siberian Husky | male | SAMN33428296 | SRR23628618 |
| D06814 | PRJNA937381 | Siberian Husky | female | SAMN33428297 | SRR23628617 |
| D06815 | PRJNA937381 | Siberian Husky | female | SAMN33428298 | SRR23628616 |
| D06816 | PRJNA937381 | Akita | female | SAMN33428299 | SRR23628615 |
| D06838 | PRJNA937381 | Boston Terrier | male | SAMN33428300 | SRR23628614 |
| D06946 | PRJNA937381 | Shiloh Shepherd | male | SAMN33428301 | SRR23628613 |
| D06969 | PRJNA937381 | Labrador Retriever | male | SAMN33428302 | SRR23628612 |
| D07204 | PRJNA448733 | Portuguese Water Dog | female | SAMN08873224 | SRR7120198 |
| D07206 | PRJNA448733 | Portuguese Water Dog | male | SAMN08873226 | SRR7120200 |
| D07208 | PRJNA448733 | Portuguese Water Dog | male | SAMN08873228 | SRR7120202 |
| D07229 | PRJNA263947 | German Shepherd | female | SAMN13655911 | SRR10752632 |
| D07231 | PRJNA233638 | German Shepherd | female | SAMN02585205 | SRR1130350 |
| D07233 | PRJNA233638 | German Shepherd | male | SAMN02585209 | SRR1132488 |
| D07238 | PRJNA192935 | German Shepherd | missing | SAMN01974494 | SRR782082 |
| D07239 | PRJNA263947 | German Shepherd | male | SAMN10940819 | SRR8614058 |
| D07240 | PRJNA543738 | German Shepherd | male | SAMN11843702 | SRR9117681 |
| D07513 | PRJNA755602 | American Staffordshire Terrier | female | SAMN20838200 | SRR15505576 |
| D07546 | PRJNA937381 | Portuguese Water Dog | male | SAMN33428303 | SRR23628611 |
| D07558 | PRJNA937381 | Portuguese Water Dog | male | SAMN33428304 | SRR23628610 |
| D07849 | PRJNA937381 | Golden Retriever | male | SAMN33428305 | SRR23628608 |
| D07887 | PRJNA937381 | German Shepherd | male | SAMN33428306 | SRR23628607 |
| D07889 | PRJNA937381 | German Shepherd | male | SAMN33428307 | SRR23628606 |
| D07938 | PRJNA937381 | Miniature Australian Shepherd | male | SAMN33428308 | SRR23628605 |
| kramer | TBD | Great Dane | male | SAM33191366 | SRR23363275 |
| D08154 | PRJNA937381 | Cavalier King Charles Spaniel | female | SAMN33444818 | SRR23628604 |
| vanna | TBD | Great Dane | female | SAM33191369 | SRR23363272 |

**Supplemental Table 1.** Samples, breeds, and SRA numbers for the 100 included in benchmarking WAGS.
